# Location of Triple-Negative Breast Cancers: Comparison with Estrogen Receptor-Positive Breast Cancers on MR Imaging

**DOI:** 10.1371/journal.pone.0116344

**Published:** 2015-01-21

**Authors:** Won Hwa Kim, Wonshik Han, Jung Min Chang, Nariya Cho, In Ae Park, Woo Kyung Moon

**Affiliations:** 1 Department of Radiology, Seoul National University Hospital, Seoul, Republic of Korea; 2 Department of Surgery, Seoul National University College of Medicine, Seoul, Korea; 3 Department of Pathology, Seoul National University College of Medicine, Seoul, Korea; University Medical Centre Utrecht, NETHERLANDS

## Abstract

There has been a major need to better understand the biological characteristics of triple-negative breast cancers. Compared with estrogen receptor (ER)-positive cancers, several magnetic resonance (MR) imaging findings have been reported as characteristic findings. However, information regarding their location has not been described. Our study was to compare the location of triple-negative breast cancers with that of ER-positive breast cancers using magnetic resonance (MR) imaging. The locations of 1102 primary breast cancers (256 triple-negative and 846 ER-positive) in 1090 women (mean, 52.1 years) were reviewed using three-dimensional (3D) coordinates. The x-axis measurement was recorded as the transverse distance from the posterior nipple line; y-axis measurement as the anteroposterior distance from the chest wall; z-axis measurement as the superoinferior distance from the posterior nipple line. The association between breast cancer subtype and tumor location was evaluated using multiple linear regression analysis. Triple-negative breast cancers were significantly closer to the chest wall than ER-positive breast cancers in absolute (1.8 cm vs. 2.3 cm, *P* < .0001) and normalized (0.21 vs. 0.25, *P* < .0001) y-axis distances. The x- and z-axes distances were not significantly different between triple-negative and ER-positive breast cancers. Multiple linear regression analysis revealed that age, mammographic density, axillary nodal status, and triple-negative subtype were significantly associated with absolute and normalized distances from the chest wall (all *P* < .05). Our results show that triple-negative breast cancers have a tendency toward a posterior or prepectoral location compared with ER-positive breast cancers.

## Introduction

Previous studies have shown that the location of a primary cancer could affect tumor growth patterns and have a prognostic significance [1]. One such study reported that patients with tumors in the medial location of the breast have worse prognosis [2, 3], and occult spread to internal mammary lymph nodes was attributed to an increased risk of relapse and breast cancer death for patients with tumors in the medial location of the breast. Moreover, research has demonstrated that lymph node metastasis occurs more commonly in tumors located in the lateral portion of the breast [4, 5]. In addition to their prognostic significance, familial breast cancers have been reported to have a preferential location. One study showed that more than half (42 of 75) of their sample of familial breast cancers resided in the posterior region of the breast, and most frequently (40 of 42 cancers) the immediate prepectoral region of the breast [6]. Furthermore, a posterior or prepectoral tumor location has been regarded as a reason for missed cancers on mammography, particularly when combined with dense parenchyma [7–9].

Recently, there has been a major need to better understand the biological characteristics of triple-negative (estrogen receptor [ER]-negative, progesterone receptor [PR]-negative, and human epidermal growth factor receptor 2 [HER2]-negative) breast cancer, which accounts for 15% to 20% of newly diagnosed breast cancer cases [10–12]. Triple-negative breast cancers have been reported to have less axillary lymph node metastasis but poorer prognosis due to distant metastasis compared with ER-positive cancers [11, 13]. The imaging and histopathologic features of triple-negative breast cancers have been described in the literature [14]. Compared with ER-positive cancers, several magnetic resonance (MR) imaging findings, such as smooth margin, rim enhancement, and intratumoral necrosis have been reported as characteristic findings [14–16]. However, information regarding their location, to our knowledge, has not been described. Our hypothesis was that triple-negative breast cancers, the most aggressive and common type of breast cancer in younger women with a family history, may have a tendency to be located in the medial or posterior region of the breast. Anatomical and lymphoscintigraphic studies have revealed that tumors in the medial and posterior locations have considerable lymphatic dissemination to the internal mammary node chain, which is the most important destination for lymph drainage outside of the axilla [17].

Thus, the aim of this study was to compare the location of triple-negative breast cancers with that of ER-positive breast cancers using MR imaging.

## Materials and Methods

### Patients

Institutional review board of Seoul National University Hospital approved our retrospective study and the requirement for informed consent was waived for this retrospective analysis. Patient record or information was anonymized and de-identified prior to analysis. Between June 2009 and May 2012, a search of a computerized MR imaging and pathology database identified 411 patients with triple-negative breast cancers and 1244 patients with ER-positive breast cancers who were diagnosed with invasive breast cancer and underwent breast MR imaging prior to surgery at our institution. HER2-enriched cancers were excluded to focus on comparing the most contrasting subtypes (triple-negative and ER-positive cancers) and simplify into two comparison groups to minimize potential statistical errors from multiple comparison. ER-negative, PR-positive, and HER2-negative cancers were also excluded for the same reasons. Among these 1655 patients, patients who received neoadjuvant chemotherapy and without pre-therapy MR imaging (n = 114), patients who underwent excisional biopsy or had prior breast surgery before MR imaging (n = 79), and those without immunohistochemistry (IHC) or fluorescence in situ hybridization (FISH) for HER2 (n = 15) were excluded. Patients with microinvasive breast cancers (n = 15), multifocal or multicentric breast cancers (n = 336), and those with breast implants (n = 6) were also excluded due to difficulty in determining the exact location of the invasive cancer. Finally, 1102 primary breast cancers (256 triple-negative and 846 ER-positive) in 1090 patients (bilateral cancers in 12 patients) comprised our study group. The mean age of the patients was 52.1 years (range, 20–84 years). A total of 432 cancers (39.2%) were detected at screening. The mean size of the tumors was 2.5 cm (range, 0.2 cm–5.9 cm). A total of 924 of the 1102 cancers (83.8%) were treated with breast-conserving surgery, and 178 cancers (16.2%) were treated with mastectomy. During this period, MR imaging was routinely performed in all patients prior to surgery for breast cancer in our institution.

### MR imaging examination

MR imaging was performed with the patient placed in a prone position. MR examinations were performed using a 1.5-T scanner (Signa; General Electric Medial Systems, Milwaukee, WI) with a dedicated breast coil (8-channel HD breast array, General Electric Medical Systems). After obtaining a bilateral transverse localizer image, sagittal fat-suppressed T2-weighted fast spin-echo images were obtained (TR/TE, variable from 5500 to 7150/82; 256×160 matrix; field of view, 200×200 mm; 1.5-mm slice thickness, no gap). Dynamic contrast-enhanced examinations included one pre-contrast and five post-contrast bilateral sagittal image acquisitions using a fat-suppressed T1-weighted three-dimensional (3D) fast spoiled gradient echo sequence (TR/TE, 6.5/2.5; 256×160 matrix; flip angle, 10°; field of view, 200×200 mm; 1.5-mm slice thickness, no gap). Gadobenate dimeglumine (0.1 mmol/kg Multihance; Bracco Imaging, Milan, Italy) was injected using an automated injector (Spectris MR, Medrad Europe, Maastricht, Netherlands) through an indwelling IV catheter. Five post-contrast image series were obtained at 76, 165, 345, 434, and 583 seconds after contrast administration. For all studies, early subtraction (i.e., first post-contrast images minus pre-contrast images), axial reformatted images, and 3D maximum intensity projection (MIP) images were generated.

### Image analysis

First, a retrospective analysis for the tumor location was performed by two radiologists in consensus (*** and *** with 6 and 15 years of experience in breast MR imaging, respectively), and they were blinded to the clinicopathologic data. To determine the location of the tumor, the whole series of MR images, including axial reformatted and MIP images, were comprehensively reviewed with a picture archiving and communication system (PACS) workstation. The tumor locations were classified as follows: 1) quadrant location (upper-outer quadrant, upper-inner quadrant, lower-outer quadrant, lower-inner quadrant, and periareolar); 2) mediolateral location (medial, central, and lateral); and 3) anteroposterior location (anterior, middle, and posterior) [18]. The mediolateral and anteroposterior locations of the breast were determined based on trisection of the breast hemisphere. If a disagreement was present in determining the tumor location, the location of the tumor center was precisely identified using imaginary lines which divide the breast into thirds.

**Figure 1:**
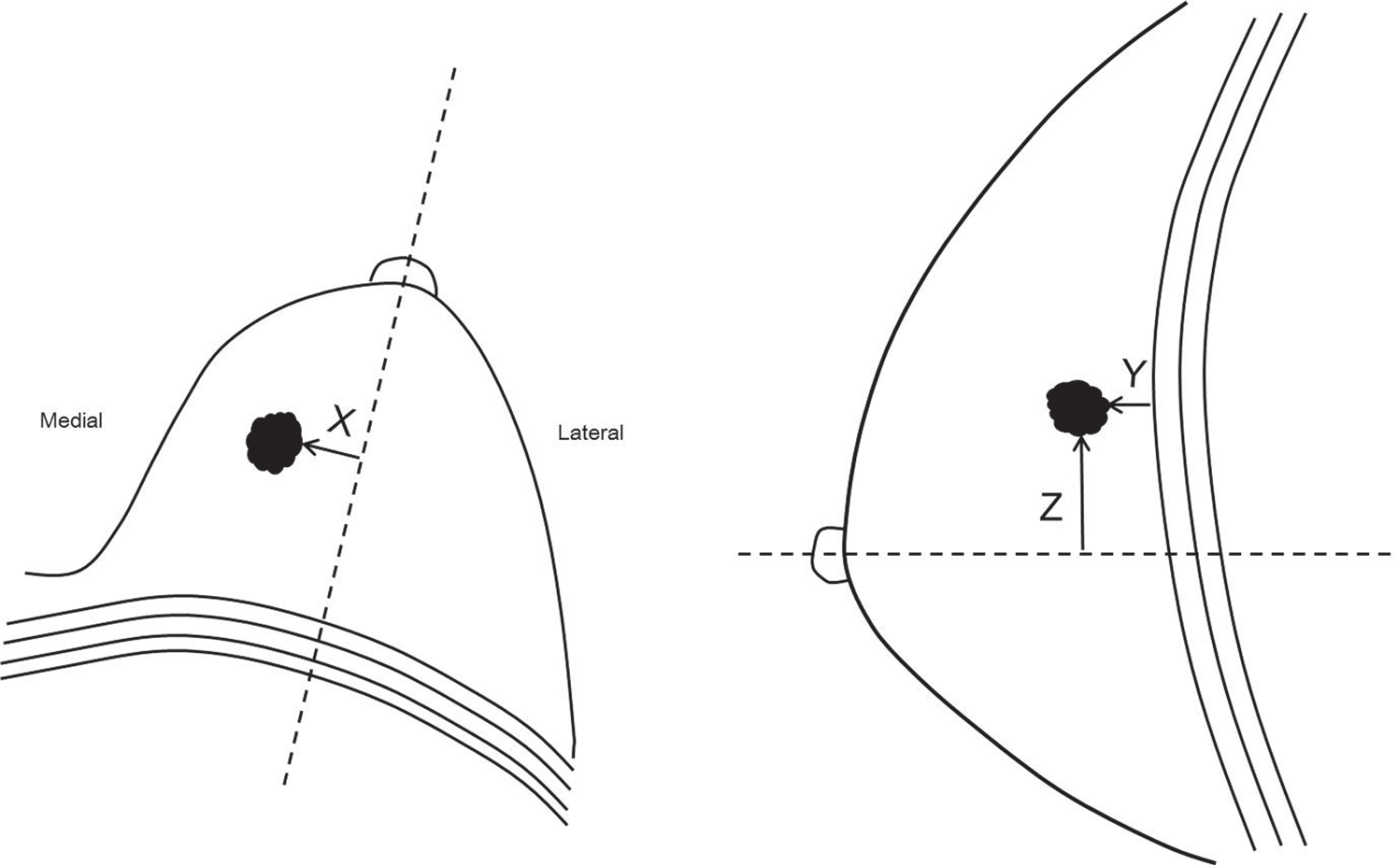
Diagram of tumor distances in the three-dimensional planes using breast MR images. Diagram shows how the distances in the x, y, and z planes were measured in axial and sagittal breast MR images. We measured the shortest distances for x-axis (X) and z-axis (Z) measurements of breast cancer in relation to the imaginary lines from the nipple and for y-axis (Y) measurement from the chest wall.

In addition, one radiologist (***, with 6 years of experience in breast MR imaging) who was not involved in subjective determination of tumor location and was also blinded to clinicopathologic data identified the tumor location using 3D coordinates (Fig. 1) [19]. In axial MR images, the x-axis measurement was recorded as the transverse distance from the posterior nipple line, an imaginary line running across the nipple back to and perpendicular to the pectoralis muscle. Tumors that were located lateral to the posterior nipple line were recorded as positive numbers, and those medial to the posterior nipple line were recorded as negative numbers. In sagittal MR images, the y-axis measurement was recorded as the anteroposterior distance from the chest wall; thus, this is equivalent to the distance from the chest wall. To account for the curvature of the chest wall, the shortest distance from the chest wall at an imaginary line that is perpendicular to the surface of the chest wall projected from the tumor was recorded. The z-axis measurement was recorded as the superoinferior distance from the posterior nipple line. Tumors that were located superior to the posterior nipple line were recorded as positive numbers and those inferior to the posterior nipple line were recorded as negative numbers. As the tumors have various morphologic features including mass and non-mass enhancement, we measured the shortest distance from the margin of the lesions rather than using center of the mass as the reference points. On MR imaging, there were 1061 mass and 41 non-mass enhancement lesions.

Because each patient has a different breast size, measurements for breast size were performed in the x-, y-, and z-axes for each patient. For the x-axis measurement, the maximum transverse dimension of the breast size was recorded; for the y-axis measurement, the anteroposterior distance from the nipple to the chest wall was recorded; and for the z-axis measurement, the craniocaudal distance from the upper edge of the breast to the lower edge was recorded. To obtain normalized x-, y-, and z-axes distances, the recorded x-, y-, and z-axes distances were divided by the corresponding breast size measurements. For example, the normalized y-axis distance equaled the y-axis distance divided by the maximum distance from the nipple to the chest wall.

### Histopathologic analysis

Histopathologic diagnoses were performed by a pathologist with 25 years of experience. The histopathologic analysis included histologic type, tumor size, histologic grade (Elston and Ellis method [20], and axillary nodal status. The routinely formalin-fixed, paraffin-embedded tissue blocks were sectioned to 4 μm thickness and then used for IHC. The expression of ER, PR, and HER2 was evaluated with the avidin-biotin complex IHC technique. ER and PR positivity was defined as the presence of 1% or more positively stained nuclei in ten high-power fields [21]. The HER2 expression was scored as 0 (no staining), 1+ (weak and incomplete membrane staining), 2+ (strong, complete membrane staining in ≤10% of tumor cells or weak/moderate heterogeneous complete staining in ≥10% of tumor cells) and 3+ (strong, complete membrane staining in >30% of tumor cells) by immunohistochemistry staining. Tumors with a score of 3+ were classified as positive for HER2 overexpression, whereas tumors with scores of 0 or 1+ were considered as negative. We performed FISH for tumors with a score of 2+ using the PathVysion HER2 DNA probe (Abbott Molecular Inc., Downers Grove, IL). HER2 expression was considered positive if the ratio of HER2 gene copies to chromosome 17 signals was greater than 2.2. The ER-positive breast cancers was defined as ER-positive, HER2-negative, and PR may be positive or negative; the triple-negative subtype as ER-negative, PR-negative, and HER2-negative tumors [22].

### Data collection and statistical analysis

The patient age, family history of breast cancer, palpability, mammographic density according to Breast Imaging Reporting and Data System (BI-RADS) [18], mammographic visibility, and histopathologic data were collected. To compare the clinicopathologic findings and location of the triple-negative and ER-positive breast cancers, the chi-square or Fisher’s exact tests, and the unpaired Student *t* test were used. For more than two groups of comparison, one-way analysis of variance (ANOVA) and Bonferroni-Dunn posthoc tests were performed. We used the chi-square test for trend (two sided) to assess whether a location trend existed among the histologic grades and mammographic breast densities. The independent relationship between absolute/normalized x-, y-, z-axes distances and tumor subtype was assessed using multiple linear regression with age, family history of breast cancer, mammographic density, tumor size, histologic grade, and axillary nodal status as potential confounding variables. A *P* value of less than .05 was considered to indicate statistical significance. All statistical analyses were performed using SPSS version 12.0 software (SPSS, Chicago, IL).

## Results

### Clinicopathologic findings

Patients with triple-negative breast cancers had more frequent family history of breast cancer (*P* = .014), palpable symptom (*P* < .0001), larger tumor size (*P* < .0001), higher histologic grade (*P* < .0001), and negative axillary nodal status (*P* = .001). Age, mammographic breast density, and mammographic visibility of the cancers were not significantly different between patients with triple-negative and ER-positive breast cancers. Table 1 demonstrates the clinicopathologic characteristics of patients with triple-negative breast cancers and those with ER-positive breast cancers.

**Table 1:** Clinicopathologic Characteristics.

**Variables**	**Triple-Negative Cancer (n = 256)**	**ER-Positive Cancer (n = 846)**	***P* value**
Age (y)^a^	51 (20 – 78)	52 (22 – 84)	.069
Family history of breast cancer	26 (10.2)	47 (5.6)	.014
Palpability			< .0001
No	60 (23.4)	379 (44.8)	
Yes	196 (76.6)	467 (55.2)	
Mammographic breast density			.934^b^
Grade 1	25 (9.8)	75 (8.9)	
Grade 2	56 (21.9)	185 (21.9)	
Grade 3	127 (49.6)	436 (51.5)	
Grade 4	48 (18.8)	150 (17.7)	
Mammographic visibility			.151
No	19 (7.4)	83(9.8)	
Yes	237(92.6)	763(90.2)	
Histologic type			
Invasive ductal	235 (91.8)	755 (89.2)	.288
Invasive lobular	0	45 (5.3)	< .0001
Mucinous	0	32 (3.8)	< .0001
Metaplastic	13 (5.1)	0	< .0001
Tubular	0	5 (0.6)	.596
Apocrine	4 (1.6)	0	.003
Adenoid cystic	2 (0.8)	0	.054
Invasive papillary	1 (0.4)	6 (0.7)	1.000
Invasive micropapillary	0	2 (0.2)	1.000
Medullary	1 (0.4)	1 (0.1)	.411
Tumor size(cm)^a^	2.2 (0.2 – 5.4)	1.9 (0.2 – 5.9)	< .0001
Histologic grade			< .0001^b^
I	2 (0.8)	125 (14.8)	
II	35 (13.7)	478 (56.5)	
III	219 (85.5)	243 (28.7)	
Axillary nodal status			.001
Negative	216 (84.4)	628 (74.2)	
Positive	40 (15.6)	218 (25.8)	
Surgery			.628
Breast-conserving surgery	212 (82.8)	712 (84.2)	
Mastectomy	44 (17.2)	134 (15.8)	

Unless otherwise indicated, data are numbers of patients and numbers in parentheses are percentages.

^a^Data are mean values, with ranges in parentheses.

^b^Chi-square test for trend.

### Distribution of tumor location

Among 1102 breast cancers, we found no significant differences in the distribution of quadrants and mediolateral locations between triple-negative and ER-positive breast cancers (Table 2). However, the distribution of the anteroposterior location was significantly different between triple-negative and ER-positive breast cancers. Triple-negative breast cancers tended to be located more posteriorly compared to ER-positive breast cancers (*P* = .0026): 54.7% (140/256) of the triple-negative breast cancers had a posterior location, whereas 43.7% (370/846) of the ER-positive breast cancers had a posterior location. When we defined ‘prepectoral’ location of tumor as the absolute distance of tumor from the chest wall less than 3mm, triple-negative breast cancers had more frequently prepectoral location than ER-positive breast cancers (17% [43/256] vs. 11% [95/846], *P* = .018).

**Table 2:** Distribution of Tumor Location.

**Variables**	**Triple-Negative Cancer (n = 256)**	**ER-Positive Cancer (n = 846)**	***P* value**
Quadrants			
Upper outer	126 (49.2)	375 (44.3)	.174
Upper inner	73 (28.5)	225 (26.6)	.574
Lower outer	26 (10.2)	112 (13.2)	.235
Lower inner	18 (7.0)	61 (7.2)	1.000
Periareolar	13 (5.1)	73 (8.6)	.064
Mediolateral location			.755^a^
Medial	51 (19.9)	186 (22.0)	
Central	100 (39.1)	310 (36.6)	
Lateral	105 (41.0)	350 (41.4)	
Anteroposterior			.0026^a^
Anterior	17 (6.6)	80 (9.5)	
Middle	99 (38.7)	396 (46.8)	
Posterior	140 (54.7)	370 (43.7)	

Data are numbers of patients and numbers in parentheses are percentages

^a^Chi-square test for trend.

Patients with tumors in the medial location were significantly younger than with tumor in the central location (*P* < .0001; posthoc analysis). Patients with tumors in the posterior location were significantly younger than those with tumors in the anterior and middle locations (all *P* < .0001; posthoc analysis). Patients with denser mammographic density tended to have tumors more posteriorly located (*P* < .0001; test for trend, dichotomized into posterior vs. anterior or middle). Tumors with positive axillary nodal status tended to be more laterally and anteriorly located (*P* = .012 and *P* = .0085; test for trend).

### Location of tumors in 3D coordinates

**Figure 2:**
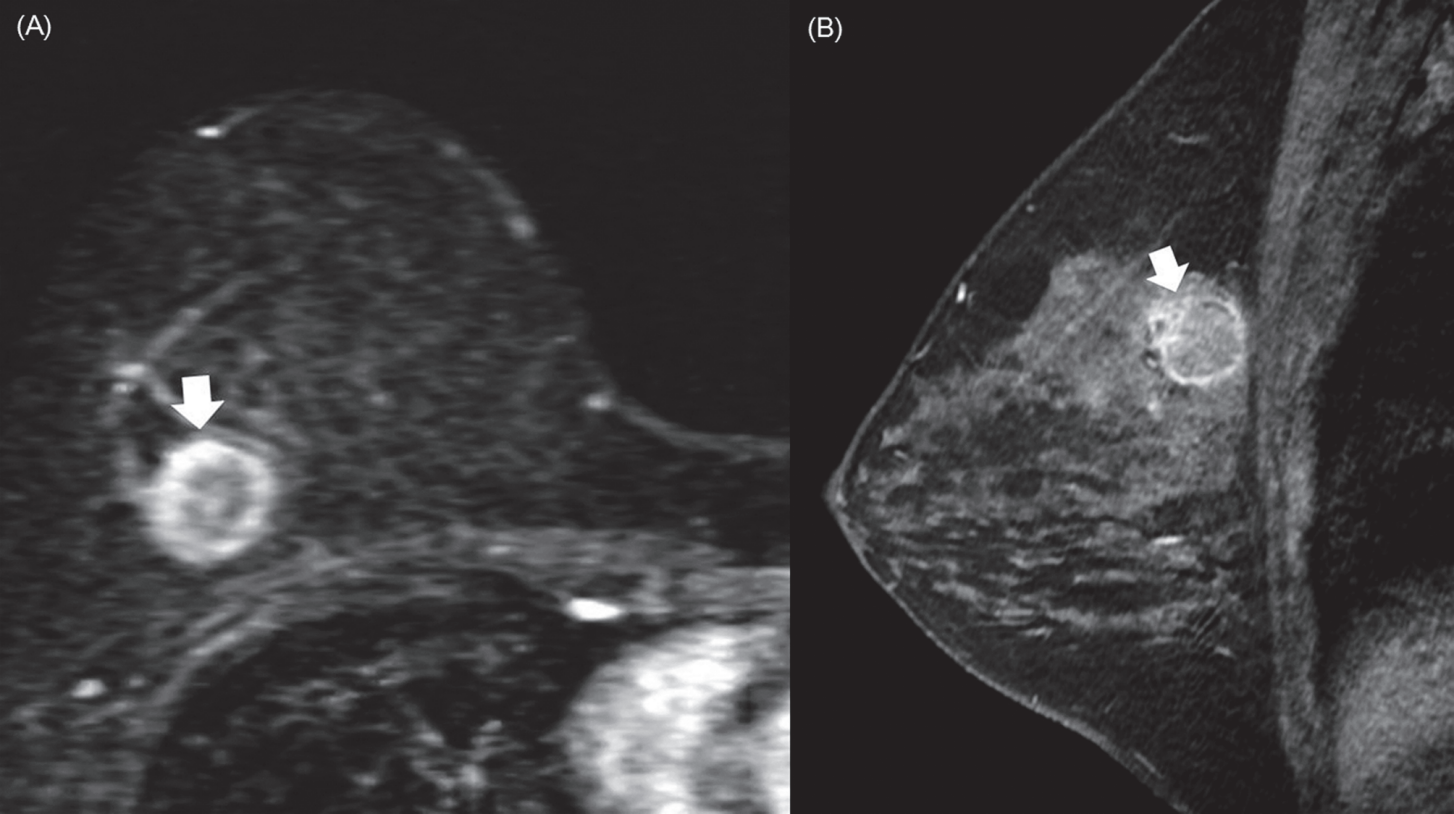
A triple-negative breast cancer in the posterior location. A triple-negative breast cancer in the posterior location of the right breast in a 41-year-old woman. (A) Sagittal early contrast-enhanced nonsubtracted T1-weighted MR image with fat suppression shows a 1.5 cm round mass (arrow) with rim enhancement. Note the tumor location in the immediate prepectoral region. (B) Mass (arrow) is also seen on subtracted axial reformatted image.

Triple-negative breast cancers were significantly closer to the chest wall (absolute y-axis distance) than ER-positive breast cancers (1.8 cm, 95% confidence interval [CI]: 1.64 cm–2.04 cm vs. 2.3 cm, 95% CI: 2.17 cm–2.43 cm; *P* < .0001) (Fig. 2). The normalized y-axis distance from the chest wall was also significantly shorter for triple-negative breast cancers compared with ER-positive breast cancers (0.21, 95% CI: 0.19–0.23 vs. 0.25, 95% CI: 0.24–0.27; *P* < .0001) (Table 3). The x- and z-axes distances were not significantly different between triple-negative and ER-positive breast cancers. Younger patients and patients with denser mammographic density tended to have tumors that were more posteriorly located (all *P* < .0001) (Tables A and B in S1 File). Tumors with higher histologic grade also tended to be more posteriorly located (*P* < .0001 and *P* = .0002 for absolute and normalized y-axes distances). Tumors with negative axillary nodal status tended to be more posteriorly located (*P* = .007 and *P* = .002 for absolute and normalized y-axes distances).

**Table 3:** Location of Tumors in 3D Coordinates.

**Parameters**	**Triple-Negative Cancer (n = 256)**	**ER-Positive Cancer (n = 846)**	***P* value**
Distance (cm)			
X-axis distance from the posterior nipple line	0.5 (0.31, 0.68)	0.4 (0.33, 0.53)	.528
Y-axis distance from the chest wall	1.8 (1.64, 2.04)	2.3 (2.17, 2.43)	< .0001
Z-axis distance from the posterior nipple line	1.2 (0.92, 1.44)	0.9 (0.78, 1.05)	.068
Normalized distance^a^			
X-axis distance from the posterior nipple line	0.06 (0.04, 0.08)	0.05 (0.04, 0.07)	.771
Y-axis distance from the chest wall	0.21 (0.19, 0.23)	0.25 (0.24, 0.27)	< .0001
Z-axis distance from the posterior nipple line	0.07 (0.06, 0.09)	0.06 (0.05, 0.07)	.087

Data are mean values, with 95% confidence intervals in parentheses.

^a^Ranges are (−0.5–0.5) for X- and Z-axes distances from the posterior nipple line and (0–1.0) for Y-axis distance from the chest wall.

Multiple linear regression analysis revealed that age, mammographic density, axillary nodal status, and triple-negative subtype were significantly correlated with both the absolute and normalized y-axis distances from the chest wall (all *P* < .05) (Table 4).

**Table 4:** Multiple Linear Regression Analysis of Association of Variables with Distance from the Chest Wall.

	**Unstandardized Coefficients**		**Standardized β Coefficient**	***P* value**
**Parameters**	**β**	**Standard Error**		
Absolute y-axis distance				
Age	0.027	0.007	0.150	< .0001
Family history of breast cancer	0.203	0.250	0.024	.418
Mammographic density	−0.511	0.082	−0.228	< .0001
Triple-negative breast cancer	−0.167	0.075	−0.073	.026
Tumor size	−0.062	0.045	−0.042	.170
Histologic grade	−0.085	0.070	−0.039	.228
Axillary nodal status	0.376	0.137	0.085	0.006
Normalized y-axis distance				
Age	0.002	0.001	0.122	.001
Family history of breast cancer	0.020	0.024	0.025	.415
Mammographic density	−0.038	0.008	−0.175	< .0001
Triple-negative breast cancer	−0.016	0.007	−0.073	.028
Tumor size	−0.009	0.004	−0.061	.049
Histologic grade	−0.009	0.007	−0.046	.172
Axillary nodal status	0.047	0.013	0.112	< .0001

## Discussion

In this study, our hypothesis was that the location of triple-negative breast cancers may have a tendency to be located in the posterior or medial region of the breast, since some of the tumors seen in high risk patients or patients with BRCA-1 gene previously reported posterior position and triple-negative breast cancers have been reported to have less axillary lymph node metastasis but poorer prognosis due to distant metastasis compared with ER-positive cancers. Our results show that triple-negative breast cancers have a tendency toward posterior and prepectoral locations on MR imaging compared to ER-positive breast cancers. The frequent association of triple-negative breast cancers with a posterior or prepectoral location and rapid tumor growth rate may explain why triple-negative breast cancers often present as interval cancers on mammography [23]. There was, however, no difference in the distribution of quadrants and mediolateral locations between triple-negative and ER-positive breast cancers. We also found that age, mammographic breast density, and axillary nodal status are associated with tumor location. Younger patients and patients with denser mammographic density tended to have tumors that were more posteriorly located (all *P* < .0001).

Schrading and Kuhl investigated imaging features in women with familial risk and found that the imaging phenotypes of cancers differ among risk categories [6]. Compared with breast cancers in women with moderate familial risk, breast cancers in women with high familial risk and BRCA1 mutation carriers often exhibit benign morphologic features (noncalcified mass with an oval shape and smooth margin) and were located in the posterior or prepectoral region of the breast. However, these authors did not analyze the results according to tumor subtype. Our results may account for their observation that BRCA1-associated breast cancers more frequently had a posterior location because BRCA1 mutation carriers have a greater tendency to have triple-negative cancers [24, 25].

Most published research regarding tumor location has utilized mammograms or clinical examinations, and has assessed the two-dimensional (2D) plane with respect to quadrants or mediolateral location [1–5]. Tumor location has rarely been investigated using the sagittal plane and anteroposterior location [6, 26]. Compared with mammography, MR imaging has many advantages for assessing the tumor location in a breast including imaging without compression, multiplanar imaging planes, and the ability to image the chest wall [27]. In addition, the sensitivity of MR imaging is higher than that of mammography, particularly in women with dense breasts [28]. These advantages are especially important when identifying the location of small cancers in the prepectoral region. To the best of our knowledge, this study is the largest series of patients in whom the location of breast cancers was assessed using MR imaging.

In our study, axillary nodal involvement was found to be less frequent in the tumors located posteriorly. As we mentioned earlier, tumors in the posterior location tended to have more non-axillary lymph node pathway than tumors in the anterior location [17]. A tendency toward posterior location in triple-negative cancers may explain that alleged clinical paradox of triple-negative breast cancers having weak relationship between tumor size, axillary nodal status, and survival [29, 30]. Our finding suggests that posterior tumor location in the triple-negative cancers might be a high risk feature for non-axillary lymph node pathway, which may need an adjuvant therapy including systemic therapy.

Our study has several limitations. First, we did not analyze all of the intrinsic subtypes of breast cancers including luminal, luminal/HER2, HER2-enriched, and basal-like subtypes [22, 31]. The definition and classification of molecular subtypes based on IHC profiles are still controversial [32]. In this study, we attempted to demonstrate an asymmetrical distribution of location according to breast cancer subtypes by comparing triple-negative and ER-positive breast cancers, which are known to be the most contrasting breast cancer subtypes. Comparison with other intrinsic subtype including luminal/HER2, HER2-enriched needs to be investigated for further research. Second, we did not analyze the results according to the triple-negative breast cancer subtype or BRCA1 mutation status. Triple-negative breast cancer is a heterogeneous disease, and the basal-like, mesenchymal-like, immune modulatory, and luminal androgen receptor subgroups have been reported [33]. Further studies are warranted to explore the impact of tumor location on the prognosis of women with triple-negative breast cancers. Third, we used two different parameters to determine tumor location. In our subjective analysis, we determined tumor location by the radiologists estimating the center of the tumor, which is similar to the clinical practice. However, using the tumor center in the objective measurement, the distance of the tumor from the reference point would be affected by the tumor size; larger tumors, particularly in prepectoral location, tend to be measured with larger distance from the chest wall. Thus, we used the margin of the tumor rather than the center of the tumor to measure the distance from the reference points. In addition, intra-observer reproducibility test for 3D coordinates in identifying tumor location was not performed in our study. However, most of our study cases were masses (96.3%) and have discrete margins.

Lastly, we did not provide mechanisms for the skewed location toward the posterior and prepectoral breast in triple-negative breast cancers. Hypothetical explanation for our observation is that posterior or prepectoral location may have a favorable environment for developing triple-negative breast cancers. The idea that microenvironment shapes the course of carcinogenesis, and hence breast cancer subtype has been discussed [34, 35]. It is interesting to note that age and mammographic density are also associated with posterior location of the tumor in our study. In various tumors, a correlation between distinctive tumor location and genetic signatures or clinical behaviors has been reported [36–38]. Further biological and radiogenomic studies are needed to explain the relationship between distinctive locations and breast cancer subtypes.

In conclusion, triple-negative breast cancers have a tendency toward a posterior or prepectoral location on MR imaging compared with ER-positive breast cancers. Special attention should be paid to these regions of the breast to detect biologically aggressive triple-negative breast cancers.

## Supporting Information

S1 File This file contains supporting Tables A and B. Table A. Distances of Tumors in 3D Coordinates According to the Clinicopathologic Features
Table B. Normalized Distances of Tumors in 3D Coordinates According to the Clinicopathologic Features
(DOC)
