# Supplementary material for: Location of Triple-Negative Breast Cancers: Comparison with Estrogen Receptor-Positive Breast Cancers on MR Imaging
Source: PLoS One. 2015 Jan 21;10(1):e0116344. doi: 10.1371/journal.pone.0116344 (PMC4301951; doi:10.1371/journal.pone.0116344)
Supplement: S1 File — Table A. Distances of Tumors in 3D Coordinates According to the Clinicopathologic Features Table B. Normalized Distances of Tumors in 3D Coordinates According to the Clinicopathologic Features (DOC) [file pone.0116344.s001.doc]

**Table A. Distances of Tumors in 3D Coordinates According to the Clinicopathologic Features**

| Variables | X-axis Distance | *P* value | Y-axis Distance | *P* value | Z-axis Distance | *P* value |
| --- | --- | --- | --- | --- | --- | --- |
| Age (y) |  | .097 |  | < .0001^a^ |  | .405 |
| 20 - 39 | 0.48 (0.19, 0.79) |  | 1.39 (1.13, 1.64) |  | 0.8 (0.38, 1.20) |  |
| 40 - 60 | 0.50 (0.39, 0.61) |  | 2.04 (1.92, 2.17) |  | 1.0 (0.88, 1.18) |  |
| ≥ 60 | 0.27 (0.09, 0.45) |  | 3.03 (2.76, 3.30) |  | 0.9 (0.67, 1.14) |  |
| Family history of breast cancer |  | .596 |  | .670 |  | .015 |
| No | 0.44 (0.35, 0.54) |  | 2.19 (2.08, 2.31) |  | 1.00 (0.87, 1.12) |  |
| Yes | 0.34 (-0.04, 0.71) |  | 2.29 (1.79. 2.80) |  | 0.34 (-0.17, 0.86) |  |
| Palpability |  | .342 |  | < .0001 |  | .346 |
| No | 0.50 (0.37, 0.62) |  | 2.57 (2.39, 2.75) |  | 0.90 (0.73, 1.08) |  |
| Yes | 0.41 (0.29, 0.54) |  | 1.94 (1.81, 2.08) |  | 1.02 (0.86, 1.18) |  |
| Mammographic breast density |  | .191 |  | < .0001^a^ |  | .587 |
| Grade 1 | 0.23 (-0.07, 0.52) |  | 3.50 (3.04, 3.95) |  | 0.77 (0.37, 1.17) |  |
| Grade 2 | 0.45 (0.26, 0.65) |  | 2.81 (2.55, 3.06) |  | 0.89 (0.66, 1.13) |  |
| Grade 3 | 0.42 (0.30, 0.54) |  | 1.97 (1.84, 2.11) |  | 1.01 (0.84, 1.18) |  |
| Grade 4 | 0.62 (0.38, 0.85) |  | 1.41 (1.20, 1.62) |  | 1.06 (0.74, 1.38) |  |
| Histologic type |  | .457 |  | .595 |  | .933 |
| Invasive ductal | 0.42 (0.33, 0.52) |  | 2.20 (2.09, 2.32) |  | 1.00 (0.88. 1.13) |  |
| Invasive lobular | 0.60 (0.18. 1.02) |  | 2.40 (1.87, 2.93) |  | 0.55 (-0.07, 1.17) |  |
| Mucinous | 0.69 (0.04, 1.33) |  | 1.89 (1.14, 2.65) |  | 0.69 (-0.13, 1.50) |  |
| Metaplastic | 0.01 (-1.32, 1.33) |  | 1.64 (1.13, 2.15) |  | 0.87 (-0.58, 2.32) |  |
| Tubular | 1.28 (0.12, 2.44) |  | 1.20 (0.12 , 2.28) |  | 2.38 (-0.01, 4.77) |  |
| Apocrine | 0.90 (-0.11, 1.91) |  | 2.80 (-0.85, 6.45) |  | -0.68 (-2.19, 0.84) |  |
| Adenoid cystic | 0.15 (-14.46, 14.76) |  | 1.75 (-5.24, 8.74) |  | 1.20 (-1.34, 3.74) |  |
| Invasive papillary | 1.14 (-0.37, 2.66) |  | 2.96 (0.83, 5.09) |  | 0.59 (-1.90, 3.08) |  |
| Invasive micropapillary | 1.55 (0.91, 2.19) |  | 1.50 (-15.01, 18.02) |  | 1.20 (-15.32, 17.72) |  |
| Medullary | 0.60 (-19.73, 20.93) |  | 0.55 (-5.17, 6.27) |  | 1.35 (-29.78, 32.48) |  |
| Tumor size^*^ |  | .630 |  | .672 |  | .547 |
| ≤ 2cm | 0.43 (0.29, 0.56) |  | 2.30 (2.12, 2.47) |  | 0.92 (0.73, 1.10) |  |
| > 2cm | 0.47 (0.34, 0.60) |  | 2.12 (1.97, 2.28) |  | 1.00 (0.82, 1.17) |  |
| Histologic grade |  | .371 |  | <.0001 |  | .351 |
| I | 0.60 (0.35, 0.86) |  | 2.39 (2.08, 2.69) |  | 1.22 (0.90, 1.54) |  |
| II | 0.45 (0.32, 0.58) |  | 2.38 (2.20, 2.56) |  | 0.95 (0.78, 1.12) |  |
| III | 0.39 (0.25, 0.53) |  | 1.93 (1.77, 2.09) ^b^ |  | 0.93 (0.73, 1.13) |  |
| Axillary nodal status |  | .002 |  | .007 |  | .020 |
| Negative | 0.34 (0.26, 0.47) |  | 2.10 (1.98, 2.23) |  | 1.06 (0.92, 1.20) |  |
| Positive | 0.69 (0.52, 0.86) |  | 2.49 (2.24, 2.74) |  | 0.72 (0.47, 0.97) |  |
| Surgery |  | .0047 |  | .0003 |  | .<.0001 |
| Breast-conserving surgery | 0.50 (0.40, 0.60) |  | 2.10 (1.99, 2.22) |  | 1.10 (0.97, 1.24) |  |
| Mastectomy | 0.15 (-0.04, 0.35) |  | 2.65 (2.36, 2.95) |  | 0.31 (0.05, 0.57) |  |

Note.― Data are mean values, with 95% confidence intervals in parentheses.

^a^*P* < .05 between all subgroups.

^b^*P* < .05 vs. grades I and II.

**Table B. Normalized Distances of Tumors in 3D Coordinates According to the Clinicopathologic Features**

| Variables | Normalized  X-axis Distance | *P* value | Normalized  Y-axis Distance | *P* value | Normalized  Z-axis Distance | *P* value |
| --- | --- | --- | --- | --- | --- | --- |
| Age (y) |  | .154 |  | < .0001^a^ |  | .645 |
| 20 - 39 | 0.05 (0.02, 0.09) |  | 0.18 (0.150 – 0.21) |  | 0.05 (0.03, 0.08) |  |
| 40 - 60 | 0.06 (0.05, 0.07) |  | 0.23 (0.22 – 0.24) |  | 0.06 (0.06, 0.07) |  |
| ≥ 60 | 0.03 (0.02, 0.06) |  | 0.31 (0.28 – 0.33) |  | 0.06 (0.04, 0.07) |  |
| Family history of breast cancer |  | .558 |  | .861 |  | .024 |
| No | 0.04 (0.04, 0.07) |  | 0.24 (0.23, 0.25) |  | 0.06 (0.06, 0.07) |  |
| Yes | 0.04 (-0.00, 0.08) |  | 0.25 (0.21, 0.30) |  | 0.04 (-0.01, 0.06) |  |
| Past history of breast cancer |  | .051 |  | .422 |  | .382 |
| No | 0.06 (0.05, 0.07) |  | 0.24 (0.23, 0.25) |  | 0.06 (0.05, 0.07) |  |
| Yes | -0.01 (-0.08, 0.05) |  | 0.27 (0.20, 0.35) |  | 0.08 (0.03, 0.14) |  |
| Palpability |  | .712 |  | < .0001 |  | .436 |
| No | 0.06 (0.04, 0.07) |  | 0.28 (0.26, 0.30) |  | 0.06 (0.05, 0.07) |  |
| Yes | 0.05 (0.04, 0.07) |  | 0.22 (0.20, 0.23) |  | 0.06 (0.05, 0.08) |  |
| Mammographic breast density |  | .041 |  | < .0001^a^ |  | .417 |
| Grade 1 | 0.02 (-0.01, 0.05) |  | 0.34 (0.30, 0.38) |  | 0.05 (0.02, 0.08) |  |
| Grade 2 | 0.05 (0.03, 0.07) |  | 0.29 (0.27, 0.31) |  | 0.06 (0.04, 0.07) |  |
| Grade 3 | 0.05 (0.04, 0.07) |  | 0.23 (0.22, 0.25) |  | 0.06 (0.05, 0.07) |  |
| Grade 4 | 0.08 (0.05, 0.11) |  | 0.17 (0.15, 0.20) |  | 0.07 (0.05, 0.09) |  |
| Pathologic type |  | .555 |  | .414 |  | .787 |
| Invasive ductal | 0.05 (0.04, 0.06) |  | 0.24 (0.23, 0.25) |  | 0.06 (0.06, 0.07) |  |
| Invasive lobular | 0.08 (0.03, 0.13) |  | 0.28 (0.22, 0.34) |  | 0.02 (-0.02, 0.06) |  |
| Mucinous | 0.09 (0.01, 0.16) |  | 0.22 (0.14, 0.29) |  | 0.05 (-0.01, 0.10) |  |
| Metaplastic | 0.01 (-0.13, 0.14) |  | 0.19 (0.13, 0.25) |  | 0.04 (-0.06, 0.14) |  |
| Tubular | 0.14 (-0.00, 0.28) |  | 0.18 (-0.00, 0.36) |  | 0.14 (-0.00, 0.28) |  |
| Apocrine | 0.13 (-0.03, 0.28) |  | 0.33 (-0.07, 0.72) |  | -0.05 (-0.14, 0.04) |  |
| Adenoid cystic | 0.00 (-1.27, 1.27) |  | 0.15 (-0.49, 0.79) |  | 0.10 (0.10, 0.10) |  |
| Invasive papillary | 0.13 (-0.02, 0.28) |  | 0.29 (0.11, 0.46) |  | 0.04 (-0.11, 0.19) |  |
| Invasive micropapillary | 0.20 (0.20, 0.20) |  | 0.15 (-1.76, 2.06) |  | 0.10 (-1.17, 1.37) |  |
| Medullary | 0.05 (-1.86, 1.96) |  | 0.10 (-1.17, 1.37) |  | 0.10 (-1.17, 1.37) |  |
| Tumor size^*^ |  | .767 |  | .634 |  | .455 |
| ≤ 2cm | 0.05 (0.04, 0.07) |  | 0.25 (0.24, 0.27) |  | 0.06 (0.05, 0.07) |  |
| > 2cm | 0.06 (0.04, 0.07) |  | 0.23 (0.22, 0.25) |  | 0.06 (0.05, 0.08) |  |
| Histologic grade |  | .200 |  | .002 |  | .252 |
| I | 0.08 (0.05, 0.11) |  | 0.27 (0.24, 0.30) |  | 0.08 (0.06, 0.10) |  |
| II | 0.05 (0.04, 0.07) |  | 0.26 (0.24, 0.28) |  | 0.07 (0.05, 0.77) |  |
| III | 0.05 (0.03, 0.63) |  | 0.22 (0.20, 0.24) ^b^ |  | 0.06 (-0.05, 0.73) |  |
| Axillary nodal status |  | .003 |  | .002 |  | .011 |
| Negative | 0.05 (0.03, 0.06) |  | 0.23 (0.22, 0.24) |  | 0.07 (0.06, 0.08) |  |
| Positive | 0.08 (0.06, 0.10) |  | 0.28 (0.25, 0.30) |  | 0.04 (0.03, 0.06) |  |
| Surgery |  | .002 |  | < .0001 |  | < .0001 |
| Breast-conserving surgery | 0.06 (0.05, 0.07) |  | 0.23 (0.22, 0.24) |  | 0.07 (0.06, 0.08) |  |
| Mastectomy | 0.02 (-0.00, 0.04) |  | 0.30 (0.27, 0.33) |  | 0.02 (0.00, 0.03) |  |

Note.― Data are mean values, with 95% confidence intervals in parentheses. Ranges are (-0.5 – 0.5) for X- and Z-axes distances from the posterior nipple line and (0 – 1.0) for Y-axis distance from the chest wall.

^a^*P* < .05 between all subgroups.

^b^*P* < .05 vs. grades I and II.
